# A case report of myocardial infarction with non-obstructive coronary arteries caused by severe myocardial bridging: insights from resting full-cycle ratio and perfusion imaging

**DOI:** 10.1093/ehjcr/ytag673

**Published:** 2026-09-08

**Authors:** Wei Gao, Albert Sudharsono, Guobing Liu, Chenguang Li, Feng Zhang

**Affiliations:** Department of Cardiology, Zhongshan Hospital, Fudan University, Fenglin Road 180, Xuhui District, 200030, Shanghai, China; Department of Cardiology and Vascular Medicine, Dr Agoesdjam General Hospital, DI Panjaitan No.51, Sampit, Delta Pawan, Ketapang District, 7881, West Borneo, Indonesia; Department of Nuclear Medicine, Zhongshan Hospital, Fudan University, Fenglin Road 180, Xuhui District, 200030, Shanghai, China; Department of Cardiology, Zhongshan Hospital, Fudan University, Fenglin Road 180, Xuhui District, 200030, Shanghai, China; Department of Cardiology, Zhongshan Hospital, Fudan University, Fenglin Road 180, Xuhui District, 200030, Shanghai, China

**Keywords:** Case report, MINOCA, Myocardial bridging, Resting full-cycle ratio, Multimodality imaging

## Abstract

**Background:**

Myocardial bridging (MB) is often considered a benign anatomical variant, yet deep or long bridges can result in dynamic coronary compression and ischaemia. In rare instances, MB may lead to myocardial infarction with non-obstructive coronary arteries (MINOCA), posing a diagnostic challenge.

**Case summary:**

We report the case of a 59-year-old man who presented with chest pain and elevated cardiac troponin following exertion. Coronary angiography showed no obstructive coronary artery disease but revealed an MB in the mid-left anterior descending (LAD) artery with complete systolic compression. Intravascular ultrasound (IVUS) confirmed the presence of MB without underlying atherosclerosis. Functional assessment using the resting full-cycle ratio (RFR) demonstrated a value of 0.91. Single-photon emission computed tomography (SPECT) revealed no perfusion defects, supporting the absence of significant ischaemia. The patient was diagnosed with MINOCA secondary to MB and treated conservatively with beta-blockers. He remained free of recurrent myocardial infarction, and the exertional chest pain was markedly improved.

**Discussion:**

This case highlights the potential of severe MB to cause infarction in the absence of fixed coronary lesions. It also underscores the diagnostic utility of RFR in assessing MB physiology and its concordance with perfusion imaging.

Learning points

- Myocardial bridging, although often benign, can be an underrecognized cause of MINOCA.
- Resting full-cycle ratio is a valuable tool for functional assessment of MB and correlates well with perfusion imaging.

## Introduction

Myocardial bridging (MB) is a congenital coronary anomaly, in which a segment of an epicardial coronary artery, most commonly the mid-left anterior descending artery (LAD), tunnels through the myocardium. Although traditionally considered benign, haemodynamically significant MB has been demonstrated to be associated with angina, arrhythmia, and even myocardial infarction, particularly when deep systolic compression occurs.^1^ Myocardial infarction with non-obstructive coronary arteries (MINOCA) is a heterogeneous clinical condition and includes aetiologies such as coronary vasospasm, thrombosis, microvascular dysfunction, and MB.^2^ Here, we report a case of MINOCA caused by severe MB with complete systolic LAD compression.

## Summary figure

**Table:** 

Time	Event
7 days prior to admission	Onset of chest pain following intense physical exertion, diagnosed with non–ST-segment elevation myocardial infarction
Admission (day 1)	Asymptomatic. Repeat electrocardiogram and high-sensitivity troponin T levels were within normal limits. Chest radiography and transthoracic echocardiography revealed no abnormalities.
Day 2	Coronary angiography showed no stenosis; myocardial bridging in mid-left anterior descending. Intravascular ultrasound confirmed no plaque erosion, rupture, or thrombus. Resting full-cycle ratio was 0.91, indicating no ischaemia.
Day 3	Single-photon emission computed tomography performed; no evidence of myocardial ischaemia.
Day 5	Discharged home
3-year follow-up	Remained free of recurrent myocardial infarction and the exertional chest pain was markedly improved.

## Case Presentation

A 59-year-old male patient was referred to our centre with a diagnosis of non–ST-segment elevation myocardial infarction (NSTEMI). One week prior to admission, he experienced chest pain following intense physical exertion. The chest pain was described as a squeezing sensation accompanied by mild diaphoresis, lasted approximately 30 min, and resolved spontaneously. At the time of the event, the patient reported no emotional stress, fever, cough, or sputum production, and denied nausea, vomiting, diarrhoea, or palpitations. He subsequently presented to a local hospital, where the electrocardiogram (ECG) was largely unremarkable (*Figure 1*), but the cardiac troponin I level was markedly elevated (7.3 ng/mL). Based on these findings, the patient was diagnosed with NSTEMI and was advised to undergo coronary angiography. However, he declined the recommendation at the local hospital and presented to our institution 1 week later for further evaluation and management.

**Figure 1:**
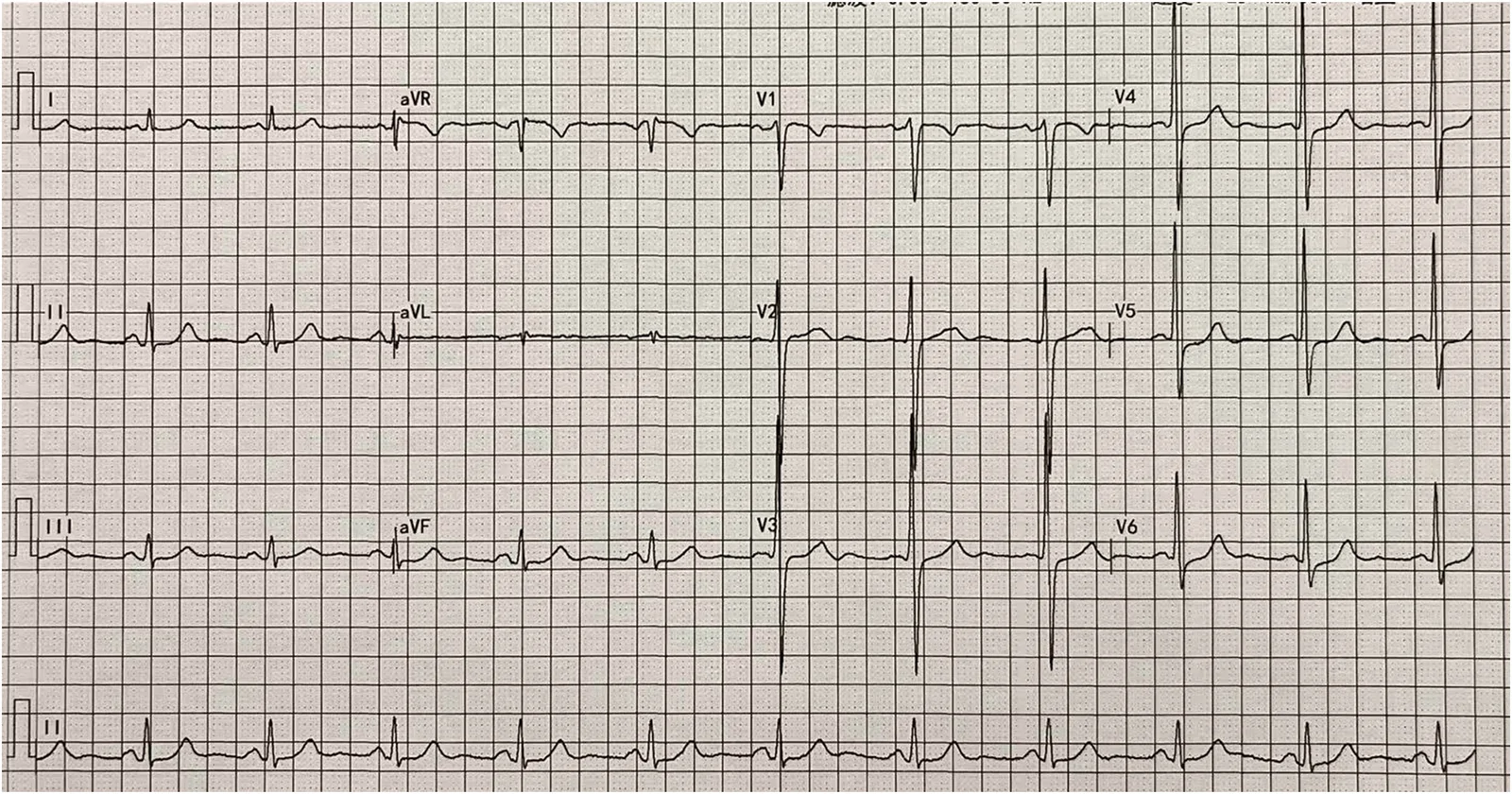
Patient’s electrocardiogram showing no significant ST-segment changes or T-wave abnormalities.

At the time of admission, the patient was asymptomatic. He had a medical history of well-controlled hypertension and a history of smoking, but no known history of diabetes mellitus, dyslipidaemia, or peripheral vascular disease. Neither the patient nor his family members had a prior diagnosis of coronary artery disease or MB. Repeat ECG and high-sensitivity troponin T levels were within normal limits. Chest radiography revealed no abnormalities. Transthoracic echocardiography revealed no regional wall motion abnormalities, and the left ventricular ejection fraction was 65%.

Coronary angiography was performed and showed no significant coronary artery stenosis (*Figure 2*). However, an MB was identified in the mid-segment of the LAD artery, with 100% systolic compression (*Figure 3*). Intravascular ultrasound (IVUS) was subsequently conducted, which confirmed the presence of an MB in the mid-LAD and revealed a prominent ‘half-moon’ sign (*Figure 4A* and Supplementary material online, video). No evidence of atherosclerotic plaque, intramural haematoma, dissection, or other abnormalities was observed throughout the LAD.

**Figure 2:**
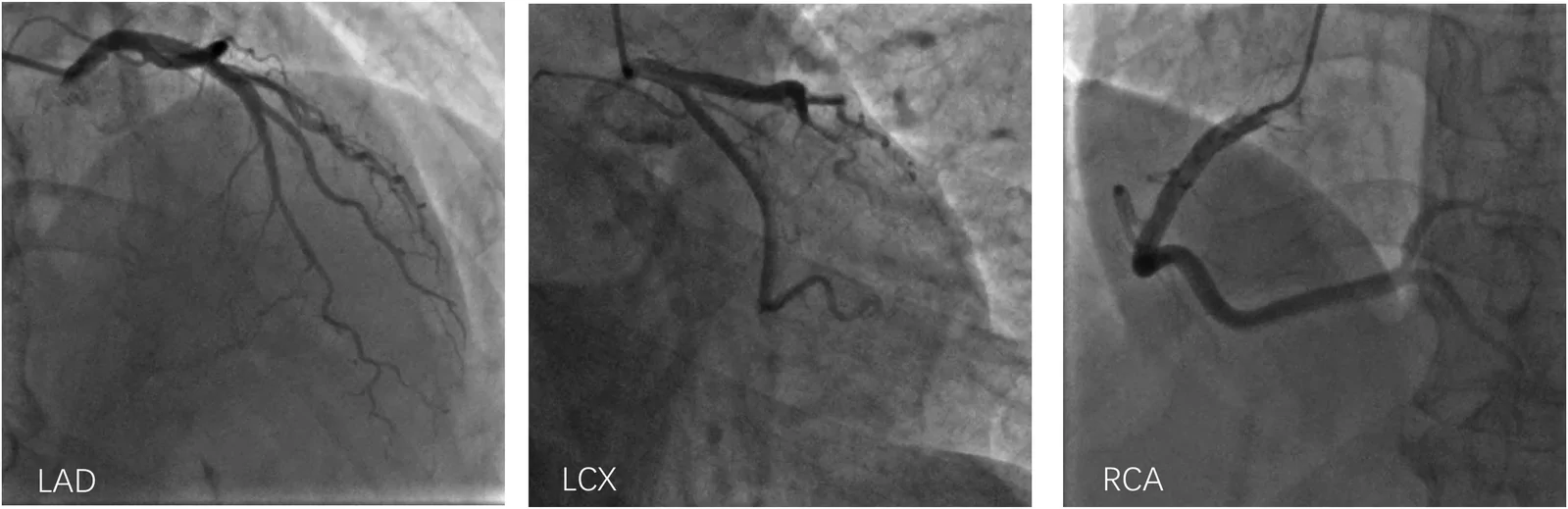
Coronary angiography images showing no significant coronary artery stenosis in the left anterior descending, left circumflex, or right coronary artery.

**Figure 3:**
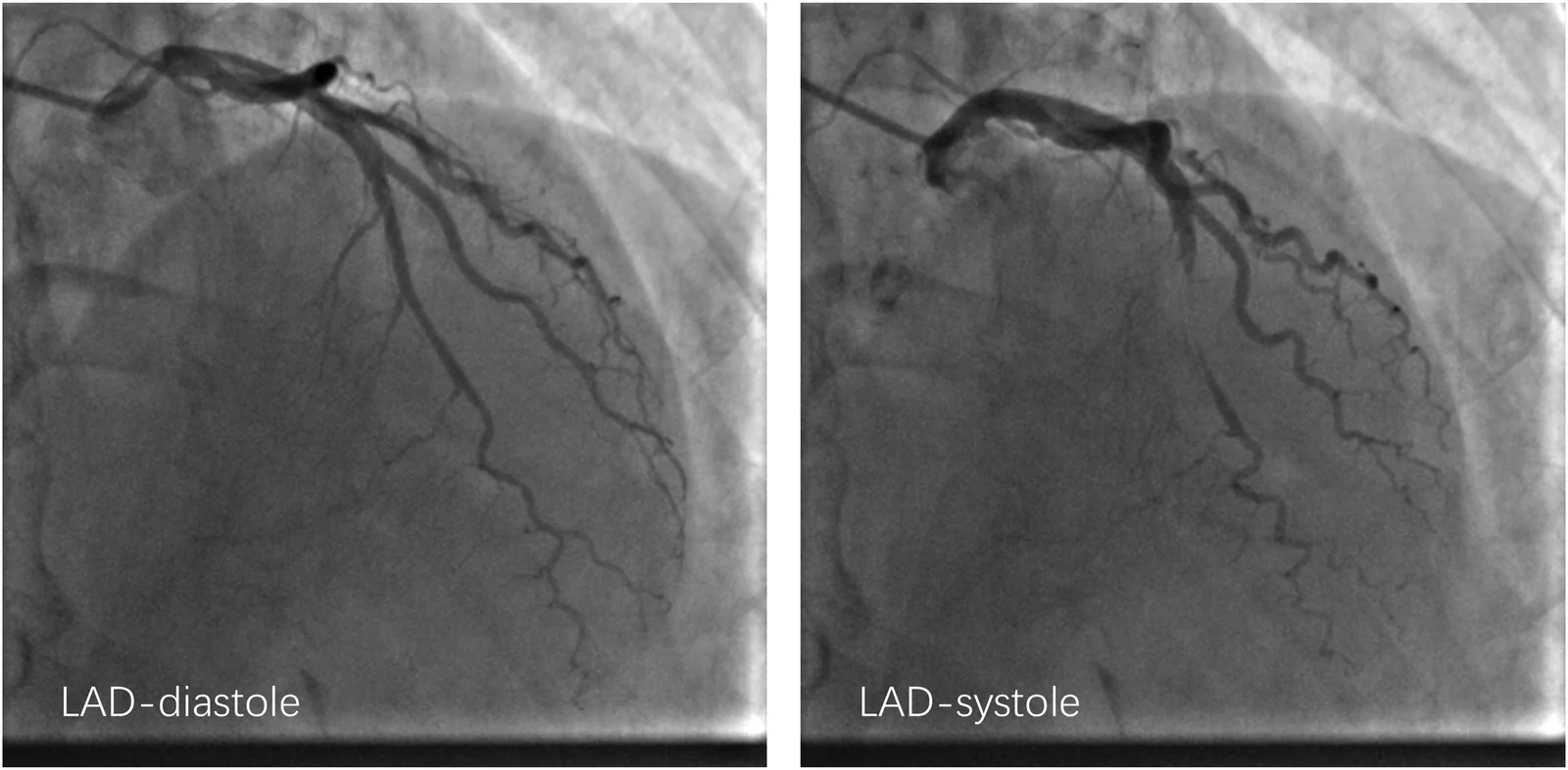
Diastolic (left) and systolic (right) frames showing complete compression of the mid-left anterior descending during systole due to myocardial bridging.

**Figure 4:**
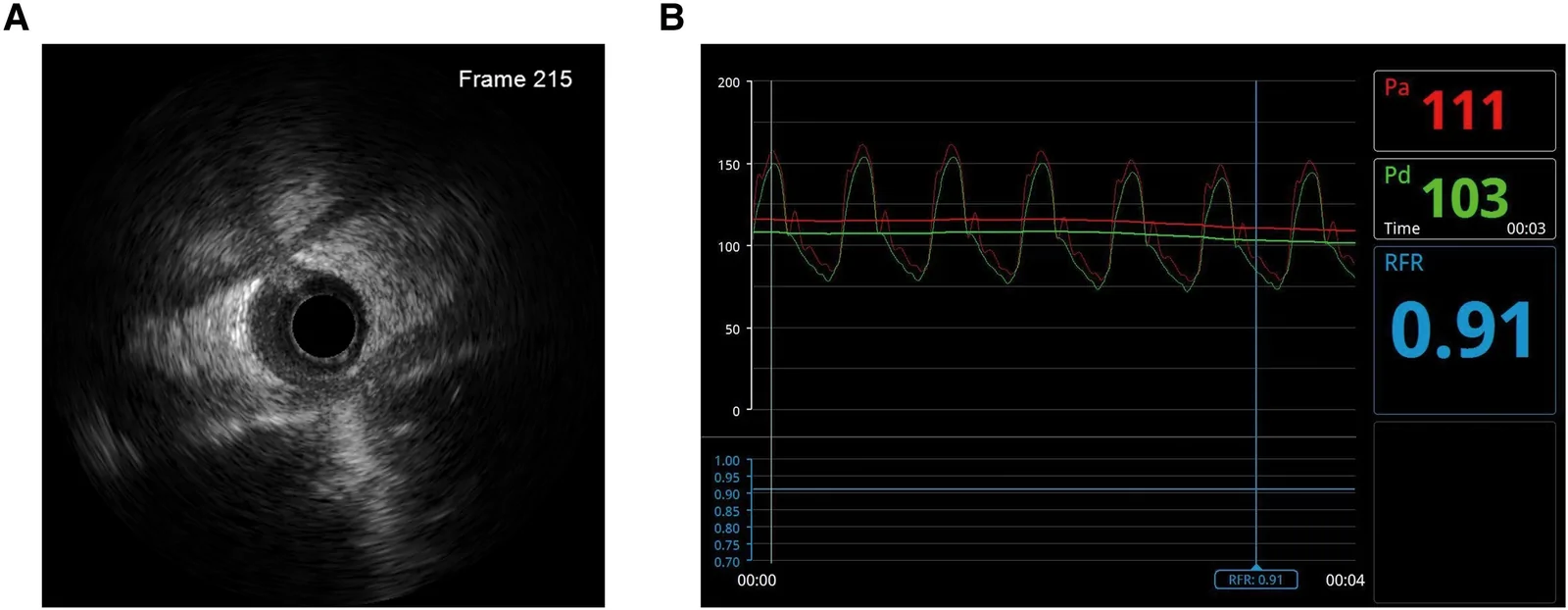
(*A*) Intravascular ultrasound image of the mid-left anterior descending demonstrating a ‘half-moon’ sign consistent with myocardial bridging and absence of plaque. (*B*) Pressure wire test showing a resting full-cycle ratio of 0.91.

Given the complete systolic compression, we assessed the physiological significance of the MB. A pressure wire was placed 15 mm distal to the bridged segment, and the RFR was measured before adenosine administration. The RFR was 0.91, indicating no evidence of significant myocardial ischaemia (*Figure 4B*). Based on these results, no interventional or surgical treatment was pursued. To further assess the presence of myocardial ischaemia, single-photon emission computed tomography (SPECT) was performed. The results revealed no significant perfusion defects at rest or during adenosine-induced stress imaging (*Figure 5A* and *B*).

**Figure 5:**
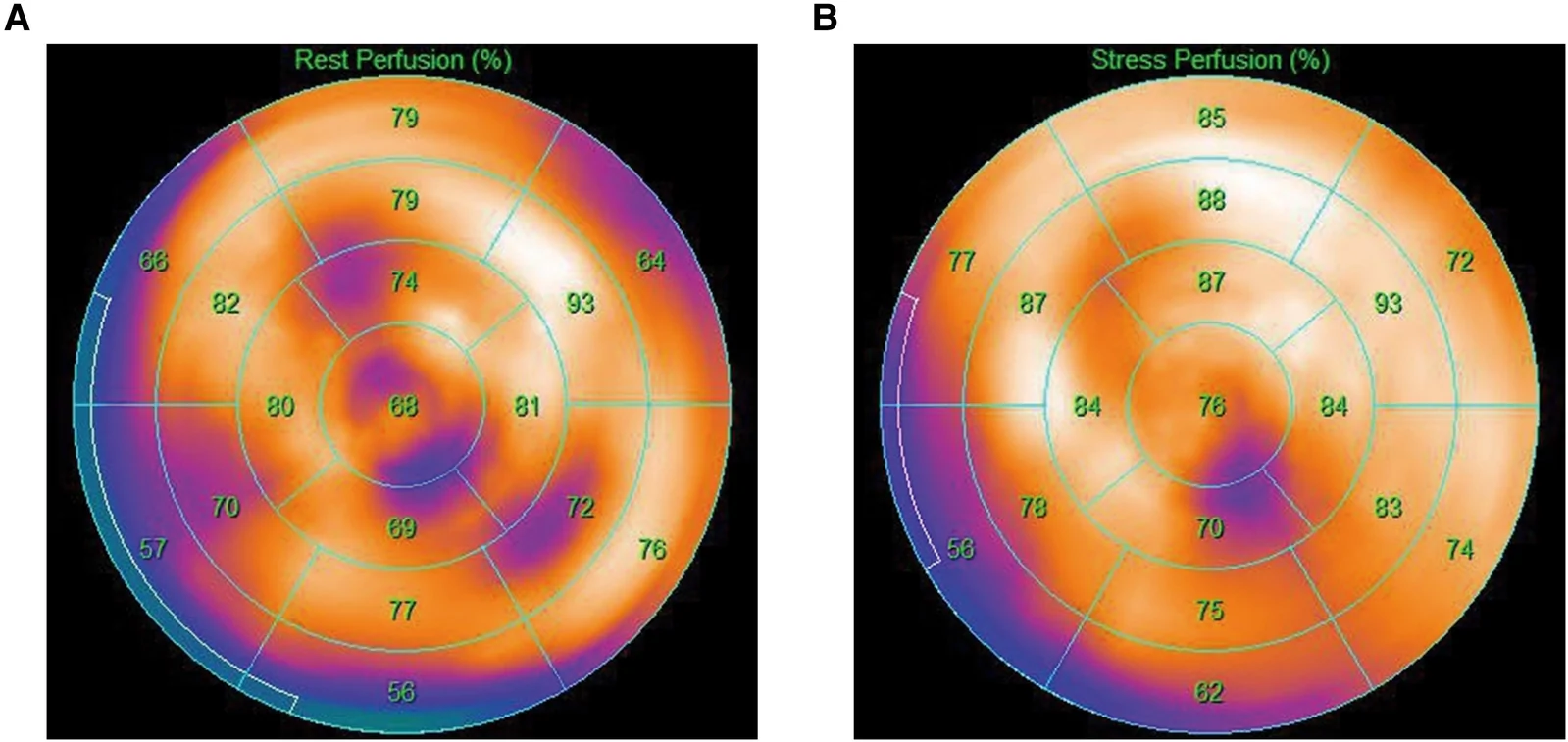
Single-photon emission computed tomography images of rest and stress perfusion showing no perfusion defect.

Considering the clinical presentation and diagnostic findings, a diagnosis of MINOCA was made, likely attributable to MB. The patient was managed conservatively with medical therapy, including aspirin 100 mg once daily and metoprolol succinate 47.5 mg once daily. After 2 days of follow-up, the electrocardiogram and cardiac biomarkers showed no significant changes compared with baseline. The patient reported no obvious chest tightness or chest pain and was subsequently discharged. During a 3-year follow-up, he remained free of recurrent myocardial infarction, and his exertional chest pain was markedly improved.

## Discussion

Although MB has long been considered a benign variant, emerging evidence indicates that haemodynamically significant MB may be associated with angina, arrhythmia, myocardial ischaemia, and even myocardial infarction in the absence of obstructive coronary disease.^3–6^ A recent review by Matta *et al*. summarized the major pathophysiological mechanisms of ischaemia in MB, including supply-demand mismatch, endothelial dysfunction and external coronary compression.^7^

The haemodynamic impact of MB may be underestimated by standard coronary angiography, as systolic compression often resolves during diastole. In our case, a 59-year-old man presented with MINOCA and was found to have 100% systolic compression of the mid-LAD due to MB, confirmed by both angiography and IVUS. Importantly, no atherosclerotic disease was detected. Functional assessment was performed using RFR, a non-hyperaemic index derived from pressure measurements across the entire cardiac cycle. The RFR value was 0.91, which falls into the so-called ‘grey zone’ where physiological assessment alone is not definitive. This highlights the necessity of integrating multimodality evaluations, including myocardial perfusion, to comprehensively assess the haemodynamic significance of MB. Regarding SPECT, scattered perfusion heterogeneity was observed at rest (*Figure 5A*), but these findings did not conform to a coronary territorial distribution and represent true perfusion defects caused by MB. The plausible explanation is that adenosine induces a generalized vasodilation of the microcirculation, which may dilute or mask subtle regional perfusion heterogeneity.

Due to systolic pressure overshooting causing errors in mean fractional flow reserve (FFR), diastolic assessments such as diastolic FFR and instantaneous wave-free ratio provide more accurate haemodynamic evaluation and are preferred for MB assessment.^8^ Similarly, RFR is a novel non-hyperaemic index that measures the lowest diastolic Pd/Pa at rest, offering an unbiased assessment of coronary stenosis severity throughout the cardiac cycle. Several previous case reports have explored the use of RFR in MB^9–10^; however, the degree of compression caused by the bridge in those cases was not severe. In our case, the MB over the left anterior descending artery showed 100% systolic compression, and to the best of our knowledge, no similar cases have been reported to date. SPECT myocardial perfusion imaging in this case served as a non-invasive validation of the physiological indices, further supporting the absence of ischaemia. Our previous study suggests that SPECT may serve as a useful tool for the functional assessment of MB.^11^

The causes of MINOCA related to MB are highly heterogeneous, and therefore, treatment needs to be individualized. Evidence-based studies on this topic are limited.^12^ For MB-related ischaemia, β-blockers should be administered. If MB is accompanied by plaque rupture or erosion, dual antiplatelet therapy and lipid-lowering therapy (and percutaneous coronary intervention if necessary) are recommended. If coronary spasm is present, calcium channel blockers should be used. For deeper or longer MBs identified as responsible for MINOCA, surgical options may be considered.^13^ In our case, no evidence of coronary spasm was found, so metoprolol was administered. Additionally, IVUS did not reveal plaque rupture, erosion, or significant plaques, so dual antiplatelet therapy and statins were not given. Nevertheless, the patient was still diagnosed with MINOCA, and therefore, single antiplatelet therapy was cautiously prescribed.

In this case, we used a combination of coronary imaging, coronary physiology, and myocardial perfusion assessment to comprehensively evaluate the pathophysiological consequences of severe MB. To the best of our knowledge, this case adds to the limited but growing body of literature demonstrating complete systolic compression due to MB as a potential cause of MINOCA. The use of RFR alongside SPECT imaging provides a valuable example of how multimodal physiological and perfusion assessments can assist in the diagnosis and management of MB-related ischaemia. This approach highlights the unique and novel aspects of the case and aims to provide readers with comprehensive information and perspectives.

## Conclusion

This case illustrates that MB, although often considered benign, can be a causative mechanism of MINOCA in the absence of coronary stenosis. The combined use of RFR and myocardial perfusion imaging allowed for a comprehensive physiological assessment, guiding a conservative management strategy with excellent long-term outcomes. Careful functional and imaging evaluation is essential in similar presentations to avoid misdiagnosis and overtreatment.

## Lead author biography


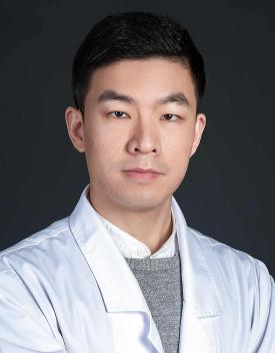


Wei Gao graduated from Fudan University in 2016 and completed his specialist training in cardiology at Zhongshan Hospital, Fudan University. He is currently engaged in interventional cardiology with a focus on coronary procedures. His main interests include coronary imaging and myocardial infarction with non-obstructive coronary arteries, and he is actively involved in related clinical research.

## Supplementary Material

ytag673_Supplementary_Data

## Data Availability

The data underlying this article will be shared on reasonable request to the corresponding author.

## Supplementary material

**Consent:** The authors confirm that written informed consent for submission and publication of this case report, including images and associated text, has been obtained from the patient in line with the Committee on Publication Ethics (COPE) guidance.
